# RTOS-Integrated Time Synchronization for Self-Deployable Wireless Sensor Networks

**DOI:** 10.3390/s26072121

**Published:** 2026-03-29

**Authors:** Sarah Goossens, Valentijn De Smedt, Lieven De Strycker, Liesbet Van der Perre

**Affiliations:** 1 WaveCore, Department of Electrical Engineering (ESAT), KU Leuven, Ghent Campus, 9000 Ghent, Belgium; lieven.destrycker@kuleuven.be (L.D.S.); liesbet.vanderperre@kuleuven.be (L.V.d.P.); 2 ECS, Department of Electrical Engineering (ESAT), KU Leuven, 2440 Geel, Belgium; valentijn.desmedt@kuleuven.be

**Keywords:** time synchronization, wireless sensor networks, FreeRTOS, LoRa, Internet of Things

## Abstract

The deployment of Wireless Sensor Networks (WSNs) remains challenging and time consuming due to the manual commissioning, configuration, and maintenance of resource-constrained Internet of Things (IoT) devices. Achieving precise network-wide time synchronization in such systems further increases this deployment complexity. This paper presents a novel Real-Time Operating System (RTOS)-integrated time synchronization method that distributes an absolute Coordinated Universal Time (UTC) reference across the network using a single Global Navigation Satellite System (GNSS)-enabled host. The method extends the semantics of the RTOS tick count by directly linking it to a global time reference. Consequently, sensor nodes obtain a notion of UTC time and can execute time-critical tasks at precisely defined moments without requiring a dedicated Real-Time Clock (RTC) or GNSS module on each sensor node. This design reduces both hardware cost and overall system complexity. Experimental results obtained on custom-developed hardware running FreeRTOS demonstrate a task synchronization error below ±30 μs between the GNSS reference and a sensor node operating at a clock frequency of 32 MHz. Such precise network-wide synchronization enables more efficient channel utilization, reduces power consumption, and improves the accuracy of both local and coordinated task execution across multiple devices in WSNs. It therefore serves as a key enabler for self-deployable WSNs.

## 1. Introduction

IoT devices are increasingly being adopted across various domains, resulting in a significant rise in the number of nodes within WSNs. The number of IoT edge devices that will be deployed is predicted to grow fast, from 5–10 billion in 2020 up to 200+ billion in 2030 [1]. However, deploying these devices is often hindered by practical challenges. In applications such as greenhouse monitoring, where parameters like temperature, humidity, and plant movement [2] must be measured, installation can be limited by time constraints and a lack of technical expertise. These factors create barriers to efficiently deploying large numbers of IoT devices.

Similarly, applications involving large-scale sensor networks face additional challenges due to the high number of sensors and the inaccessibility of certain locations. This occurs in environmental monitoring tasks such as air quality management [3], forest fire detection [4], and natural disaster prediction [5]. In these scenarios, manual deployment and configuration would be highly time-consuming and costly.

The pursuit of “plug and play” sensors has been going on for two decades to address these challenges, and more recently the concept of “fire and forget” systems has emerged in the IoT domain [6,7]. These paradigms aim to enable sensors to measure and share data autonomously after installation, without requiring further user intervention.

Time synchronization is a fundamental requirement in many WSNs. It enables precise timestamping of sensor data for event timing insights, data fusion, linking simultaneous events and identifying causal relationships. Most devices rely solely on relative timing due to energy and hardware constraints in current low-power IoT deployments. However, lightweight access to an absolute notion of UTC time would greatly improve data correlation across distributed devices [8], interoperability between independent networks, and overall application reliability. UTC is the international time standard and enables consistent timestamping across distributed systems and time zones.

Beyond timestamping, synchronization is also critical for communication scheduling and is therefore a key enabler of self-deployable WSNs. A shared notion of time allows sensor nodes to autonomously coordinate communication and task execution without requiring manual configuration during deployment.

In Time Division Duplex (TDD) communication networks, synchronization ensures orderly switching between uplink and downlink on the same frequency channel. In Time Division Multiple Access (TDMA)-based systems, it aligns transmission slots among multiple users to reduce collisions and increase channel efficiency. In both cases, precise timing optimizes channel utilization, reduces guard intervals, and improves energy efficiency by allowing devices to conserve power during inactive periods, thus extending battery life [9]. Furthermore, accurate time synchronization enhances localization capabilities, for example in RF-acoustic indoor positioning systems [10].

Despite extensive research on time synchronization in WSNs, little research has been done on its integration with RTOSs. To the best of our knowledge, only the GNSS-based tick synchronization approach proposed in [11] and its extension in [12] have explored this direction. However, the growing adoption of RTOSs in IoT systems would benefit from built-in time synchronization mechanisms, which can improve task execution accuracy and enable synchronous operation across multiple devices without requiring a GNSS module at each device.

This paper presents a novel RTOS-integrated time synchronization method that extends the tick synchronization approach introduced in [12] to wireless sensor networks. In contrast to prior work, which synchronizes individual devices using local GNSS references, the proposed method distributes a global UTC time reference across the network from a single GNSS-enabled host and integrates it directly into the RTOS time base. By enabling the RTOS tick to represent absolute time, sensor nodes can execute time-critical tasks at precisely defined moments without requiring additional timing hardware. This approach reduces hardware complexity and cost while enabling synchronized operation across the network.

The main contributions of this work are summarized as follows:

1. Network-wide UTC distribution using a single GNSS reference. We extend GNSS-based tick synchronization to wireless sensor networks by distributing an absolute UTC reference from a single GNSS-enabled host to all nodes in the network, enabling consistent global time without requiring GNSS receivers on each device.
2. RTOS-level integration of absolute time. We integrate the distributed time reference directly into the RTOS timing infrastructure by replacing the RTOS tick count with the extended Unix epoch time in milliseconds. This allows applications to schedule tasks at precise global timestamps while removing the need for a dedicated RTC on each node.
3. Implementation and experimental validation on embedded hardware. We implement the proposed method on custom-developed hardware running FreeRTOS v10.5.1 (Amazon Web Services, Seattle, WA, USA) and experimentally demonstrate improved tick stability and network-wide task execution synchronization with an error below ±30 μs between a GNSS reference and a sensor node operating at a clock frequency of 32 MHz. The implementation of the proposed method is provided as Supplementary Software S1.

The remainder of this paper is structured as follows. Section 2 introduces the IoT context and the system architecture. Section 3 reviews existing time synchronization methods. Section 4 analyzes the challenges and opportunities of wireless time synchronization in RTOSs, and Section 5 presents the proposed RTOS-integrated synchronization method. Section 6 describes the experimental setup and presents the evaluation results, followed by a comparison with existing methods. Finally, Section 7 concludes the paper.

## 2. IoT Context and System Architecture

WSNs, comprising spatially dispersed sensor nodes dedicated to measuring various physical parameters, have become essential in a wide range of applications, from remote health management [13,14] and environmental monitoring [3] to industrial automation [15] and urban mobility improvements [16]. The introduction of the IoT has significantly expanded the functionality and scope of WSNs, enabling connectivity, data sharing, and real-time monitoring capabilities across diverse fields. Consequently, accurate time synchronization has become increasingly important in IoT networks. Precise timestamps enable coordinated sensing, support localization techniques, and facilitate synchronized communication schemes such as duty cycling, which improve overall system efficiency and reduce energy consumption [17].

**Figure 1:**
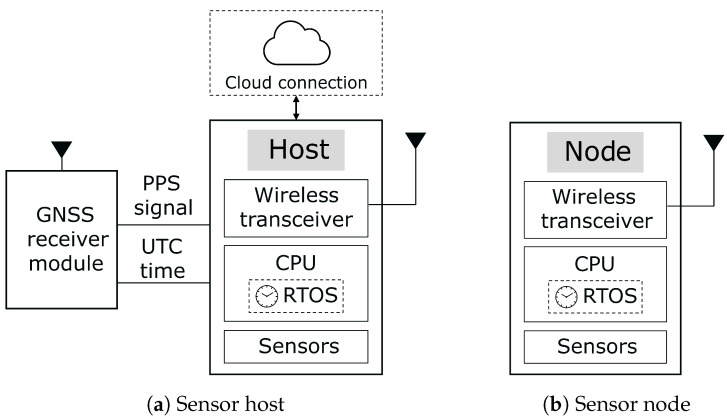
Functional architecture of the sensor host and sensor node in the proposed WSN. Both modules have a similar structure, except that the sensor host includes a GNSS module used as the global time reference for network synchronization.

We consider a sensor network architecture consisting of two module types: sensor hosts and sensor nodes, as illustrated in Figure 1a,b. Sensor hosts form a mesh network and simultaneously act as gateways for their associated sensor nodes, which connect to the host in a star topology. This hierarchical architecture provides the basis for a self-deployable WSN while also supporting the distribution of a global time reference throughout the network.

Sensor nodes are designed to operate autonomously and encapsulate relevant device information to enable automatic commissioning and integration into the network. Due to their reliance on constrained energy sources, these nodes must employ careful power management to extend operational lifetime. In addition, sensor nodes are typically designed to be cost-effective, which limits their processing and storage capabilities. Consequently, the network architecture must support lightweight mechanisms for coordination and synchronization. The functional architecture of a sensor node is shown in Figure 1b.

Sensor hosts act as access points for the sensor nodes and are connected to a GNSS module. This enables each host to determine its position and obtain an absolute UTC time reference together with the corresponding Pulse Per Second (PPS) signal. In the proposed system, this GNSS-derived reference serves as the global time source that is distributed to the sensor nodes across the network. The PPS signal is used for local tick rate compensation. The sensor host discovers new sensor nodes using a custom commissioning protocol, manages network coordination, allocates available bandwidth, and forwards collected data to the cloud. We assume the sensor hosts to be unconstrained in terms of energy consumption. Multiple hosts may coexist within the network and exchange information to manage node connectivity and data forwarding. The functional architecture of a sensor host is shown in Figure 1a.

The time synchronization method proposed in this paper has been implemented and validated using a custom-developed prototype. The hardware is based on a low-power microcontroller unit (MCU) capable of running FreeRTOS. FreeRTOS is an open-source RTOS designed for microcontrollers, providing a platform that simplifies the programming, deployment, security, connectivity, and management of small, energy-constrained edge devices [18]. Although FreeRTOS is not the most energy-efficient RTOS, it was selected for this prototype due to its widespread adoption and hence interoperability opportunities [19]. Detailed hardware information, including main component types and part numbers, is provided in Appendix A to support reproducibility of the research.

In the prototype, communication between the sensor host and the sensor node is achieved using a low-power Long Range (LoRa) 2.4 GHz RF transceiver with integrated ranging capability. This transceiver enables long-range communication in the 2.4 GHz band and maintains reliable performance in environments with heavy interference due to the inherent processing gain of the LoRa chirp spread spectrum modulation, which has been shown to provide robust operation even in the crowded 2.4 GHz industrial, scientific and medical (ISM) band [20]. LoRa at 2.4 GHz is designed for global deployment in the unlicensed ISM band, while the wider channel bandwidths available in this band enable higher data throughput compared to sub-GHz implementations.

Unlike protocols based on Carrier Sense Multiple Access with Collision Avoidance (CSMA/CA), LoRa 2.4 GHz does not employ random backoff intervals. Transmission timing is therefore not governed by channel-access backoff procedures but is primarily determined by the packet time-on-air and the host firmware that schedules packet transmissions. Consequently, the radio does not introduce additional random transmission delays. Furthermore, in contrast to sub-GHz LoRa deployments, operation in the 2.4 GHz ISM band is generally not subject to strict duty-cycle constraints.

## 3. Time Synchronization Methods

Ensuring correct timing in WSNs is challenging because in many applications distributed devices must remain closely synchronized while also coping with variable delays on wireless communication [21]. A wide range of studies has been conducted on time synchronization in WSNs, each emphasizing different aspects such as accuracy, energy efficiency, scalability, robustness, or implementation simplicity. This section describes key research contributions in this domain, including work on time synchronization mechanisms integrated within an RTOS. It further examines the advantages of embedding synchronization functionality at the operating-system level to improve timing precision and coordination across distributed nodes.

### 3.1. Existing Time Synchronization Methods for WSNs

Existing wireless synchronization solutions include Network Time Protocol (NTP), Reference Broadcast Synchronization (RBS), Timing-sync Protocol for Sensor Networks (TPSN) and Flooding Time Synchronization Protocol (FTSP). NTP is widely used for time synchronization on the Internet and typically achieves accuracy within a few milliseconds. However, the computational and energy requirements of this protocol make it unsuitable for resource-constrained IoT devices [22,23]. Consequently, more lightweight and specialized synchronization approaches are required for WSNs. RBS utilizes master broadcast reference messages, with peripherals informing others about time changes, achieving microsecond accuracy by eliminating non-deterministic delays [24]. TPSN employs handshake-based 2-way communication for synchronization with Medium Access Control (MAC) layer timestamp generation, achieving twice the accuracy of RBS [25]. FTSP reduces communication overhead by synchronizing nodes with a single radio message and adds drift compensation with MAC-layer timestamping, achieving accuracies around 1 μs [26]. These methods aim to improve time synchronization accuracy with varying approaches and precision levels. Lightweight Tree-based Synchronization (LTS) in contrast aims to minimize computation- and communication energy expended by the algorithm instead of maximizing the accuracy, by leveraging a hierarchical structure to minimize communication overhead [27]. Another approach is using a PPS signal for time synchronization. This signal can be generated by a Global Position System (GPS) to serve as the synchronization reference for multiple systems [28], or by Ultra-Wideband (UWB), resulting in a time synchronization method with sub-microsecond accuracy at the application level [29].

Despite extensive research on wireless time synchronization methods, these solutions primarily operate at the network or application layer and are not integrated into the RTOS. While these methods can synchronize clocks across nodes with high precision, they do not directly improve the predictability or coordination of task execution managed by the RTOS. Embedding time synchronization functionality within the RTOS itself can significantly enhance timing precision at the host or node level by aligning internal task scheduling with a globally synchronized time base. Combining RTOS-integrated synchronization with wireless time synchronization methods thus enables both precise local task execution and consistent network-wide timing alignment. This integration is not intended to outperform existing wireless time synchronization methods, but rather to build upon them to achieve RTOS-integrated time synchronization. As such, the approaches are complementary rather than competing.

### 3.2. Absolute Notion of Time in WSNs

Most IoT devices rely on a relative notion of time, where nodes synchronize with one another using locally maintained clocks or logical time relationships [30]. This approach is common because maintaining an absolute time reference typically requires a dedicated real-time clock together with periodic synchronization to an external source, which increases cost and energy consumption in devices that are often resource-constrained. However, access to an absolute time reference such as UTC time enables new possibilities and offers significant advantages in a wide range of IoT applications.

In industrial automation, for example, diagnostics can be scheduled at night when production is halted, minimizing downtime without disrupting real-time control. In greenhouses, lighting can be aligned with sunrise and sunset, adapting dynamically to seasonal changes while reducing energy consumption. Similarly, in satellite communication, scheduled execution ensures alignment with ground-station windows, and in medical infusion pumps, it guarantees precise drug delivery at prescribed times [31]. Additionally, in industrial IoT, the integration of time synchronization in RTOSs enables robots to perform time-critical tasks more accurately and with greater precision, which is crucial for coordinated motion control and sensing [32].

### 3.3. Time Synchronization in RTOS-Based Sensor Networks

A suitable Operating System (OS) for IoT devices must account for their typical constraints, including low power consumption, limited processing and memory resources, connectivity requirements, real-time responsiveness, and security considerations [33]. Traditional operating systems are generally not designed to meet these demands, which has led to the development of specialized OSs tailored to IoT environments [34].

Among these systems, lightweight event-driven operating systems such as Contiki and TinyOS prioritize minimal resource usage but do not provide strict real-time guarantees. In contrast, systems such as FreeRTOS, Zephyr, and RIOT incorporate real-time scheduling mechanisms that enable deterministic task execution [33,35]. Such RTOSs provide predictable timing behavior, which is essential for applications with strict latency or safety requirements, including industrial automation [36], healthcare [37], and disaster monitoring [38].

By supporting priority-based scheduling, efficient context switching, and inter-task communication mechanisms such as message queues, semaphores, and events, RTOSs enable reliable and concurrent task execution on resource-constrained devices [39,40]. Furthermore, their support for diverse hardware platforms and communication protocols facilitates the development of scalable and interoperable IoT systems [41]. Integrating time synchronization mechanisms within RTOSs further improves timing accuracy for local tasks and enables coordinated operation across distributed devices [23].

Task execution in an RTOS is influenced by temporal overheads introduced by kernel routines responsible for task scheduling. One important contributor is the context switch time, which represents the duration required to suspend one task and resume another by saving the current task’s state and restoring the next task’s state [42]. The duration of a context switch depends on factors such as the processor architecture, compiler, and system configuration, and therefore affects the predictability of task execution timing. Under specific test conditions, FreeRTOS reports a context switch time of approximately 84 CPU cycles [43].

In most RTOSs, task scheduling relies on a periodic system tick generated by a hardware timer interrupt. This interrupt is commonly produced by the SysTick timer, a processor-integrated timer that periodically triggers the scheduler. The tick occurs at a configurable rate, typically expressed in Hertz (Hz). For example, a tick rate of 1 kHz results in a tick every 1 ms. Each tick increments the system tick counter, which represents the elapsed time since system start and is used by the RTOS to manage delays, timeouts, and periodic task execution.

However, in practice, the tick rate is not perfectly constant. Oscillator inaccuracies caused by manufacturing tolerances, temperature variations, and aging introduce deviations between the nominal and actual clock frequency [44]. As a result, tasks scheduled based on the tick count may gradually drift from their intended execution time. Furthermore, even if multiple nodes in a distributed system share the same nominal tick rate, their task execution cannot remain perfectly aligned. Independent clock drift and node-specific scheduling variations, compounded by context switch overhead, inevitably introduce timing deviations between nodes over time [23]. Such drift reduces the temporal accuracy of scheduled tasks and complicates coordinated execution across multiple devices.

Previous work by Yokoyama et al. [11] and Harayama et al. [12] addresses this issue by synchronizing the RTOS tick to a GNSS PPS signal. Their method compensates for oscillator inaccuracies by adjusting the tick rate, tick phase, and system time through modifications to the SysTick timer configuration. The improved approach in [12] achieves synchronization errors below 10 μs when each microprocessor is equipped with its own GNSS receiver.

In contrast, this work extends GNSS-based RTOS synchronization to wireless sensor networks by distributing a global UTC reference from a single GNSS-enabled host to all sensor nodes. By integrating this reference into the RTOS time base and compensating for tick-rate deviations across devices, the proposed method enables more consistent task execution timing throughout the network without requiring a GNSS receiver or RTC on each node.

## 4. Time Synchronization Analysis

**Figure 2:**
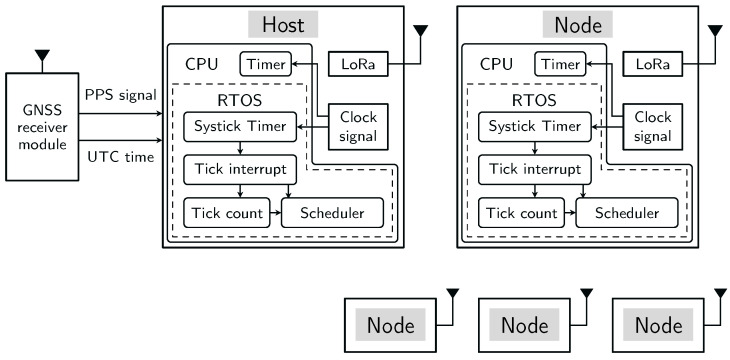
Functional architecture for synchronization procedure in exemplary setup.

During the synchronization process, the UTC time and PPS obtained from the GNSS module connected to a sensor host are used as references for establishing a common time base across the sensor network. GNSS timing provides a high-precision reference time scale, with demonstrated time transfer accuracy below 20 ns depending on the provider [45]. Sensor hosts are responsible for disseminating this time throughout the network to ensure all sensor nodes are synchronized. When a node joins the network, it receives the timing information required to align its local clock with the network-wide time reference. Figure 2 illustrates the functional structure of an exemplary setup, that was realized for experimental evaluation of the synchronization procedure. In this setup, the system comprises one host and four nodes and includes all relevant clock sources involved in the synchronization process. The host and nodes share an identical clock structure, with the host additionally connected to an external GNSS module that provides the global time reference.

Each node in a WSN maintains its own local clock. In order to achieve a shared and accurate time base across the network with the proposed time synchronization approach, the primary factors contributing to the time difference among a sensor host, a sensor node and a GNSS module are described in the following subsections.

### 4.1. Sharing of a Synchronized System Time Throughout the Network

The studies in [11,12] synchronize local system time to UTC at individual nodes using the GNSS PPS signal. This is achieved by adjusting the tick interval, i.e., the Compare Match Value of the hardware timer, until the system time aligns with the rising edge of the PPS signal. In contrast, the method proposed in this paper distributes UTC information from a single GNSS-enabled host across the sensor network, enabling all nodes to share a common time reference without requiring a GNSS module on each device.

A straightforward approach to distribute time is to use an RTC on both the host and the sensor nodes. In such a setup, the host retrieves UTC time from the GNSS module and propagates it to the nodes, which update their local RTCs. However, delays in reading and writing, as well as variations in RTC accuracy, introduce synchronization errors that accumulate across the network.

To address these limitations, the proposed method leverages the RTOS tick count to represent and propagate the extended Unix time in milliseconds. Unix time is currently defined as the number of non-leap seconds which have passed since 00:00:00 UTC on Thursday, 1 January 1970, which is referred to as the Unix epoch [46]. Instead of relying on an external RTC, the UTC time obtained from the GNSS module is converted into epoch time in seconds and multiplied by 1000 to match the millisecond resolution of the RTOS tick count. The system then replaces the tick count with this extended epoch time. Combined with the SysTick Timer count this mechanism ensures precise timekeeping across the network while maintaining an absolute notion of UTC time.

By integrating absolute time into the RTOS timing infrastructure, this method enables precise task scheduling at predefined UTC timestamps while avoiding delays associated with RTC access. Furthermore, tick rate compensation maintains timing accuracy, allowing the RTOS tick to function as a software-based RTC. This reduces hardware complexity and cost while improving synchronization performance across the network.

### 4.2. Tick Rate Error Caused by Inaccurate Crystal Oscillator

In a preemptive RTOS, e.g., FreeRTOS, ticks help determine when to switch between tasks. If time-slicing is enabled, each task runs for a set number of ticks before the scheduler switches to the next task of equal priority. The resolution of time-based operations in an RTOS is determined by the tick interval. For instance, with a 1 ms tick, the smallest measurable delay is 1 ms. However, the accuracy of the tick rate can affect the actual duration of the tick interval, as the SysTick Timer, responsible for generating tick interrupts, is subject to the inherent inaccuracies of the (crystal) oscillator used in the system.

This effect can be examined using the exemplary setup employed in this work. The system operates with a 16 MHz clock and configures the SysTick Timer to generate a 1 ms tick interval, resulting in a Compare Match Value of 15,999. Each timer count, referred to as a subtick in this article, nominally corresponds to 62.5 ns. In practice, deviations of the oscillator frequency from its nominal value introduce minor variations in the duration of each subtick, leading to tick interrupts that differ from the intended 1 ms interval.

**Figure 3:**
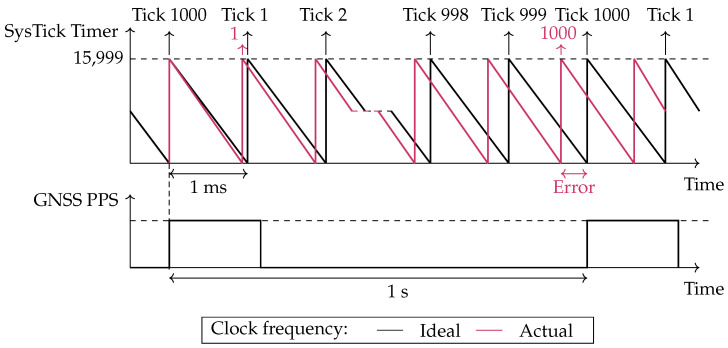
Tick rate error in an RTOS with a 16 MHz clock operating a 1 ms SysTick Timer. In this example, crystal oscillator inaccuracies cause the actual clock frequency to be slightly higher than nominal, making each tick shorter than 1 ms. Consequently, a task scheduled to run every second will gradually drift, executing at intervals slightly shorter than one second. The ideal tick rate is shown in black, and the actual tick rate in red.

This behavior is illustrated in Figure 3. The black line represents the theoretical timing of tick interrupts, where the hardware timer counts down from its maximum value to zero, generating a tick interrupt precisely every 1 ms. Assuming the first tick starts counting at a GNSS PPS pulse, exactly 1000 ticks should occur by the time the next GNSS PPS pulse arrives, marking the passage of one second. However, in reality, deviations in the subtick duration cause the tick interrupts to occur slightly faster or slower. In this example, the tick interrupts occur slightly ahead of schedule, meaning 1000 ticks are reached before the next GNSS PPS pulse, illustrated with a red line. As a result, if this tick rate is used to execute a task every second, the task will gradually drift, executing at intervals slightly shorter than one second.

Additionally, this variability in tick rates across different IoT devices complicates the synchronized execution of tasks on multiple devices, especially when each device is allocated its own communication window. Consequently, the guard interval, which ensures that transmissions do not interfere with one another, needs to be increased. Synchronizing IoT devices in time allows for more efficient use of communication windows, leading to reduced energy consumption.

As discussed in [11], the tick rate error can be compensated by adjusting the Compare Match Value of the SysTick Timer to better reflect the actual clock frequency of each individual device. In the proposed time synchronization approach, this adjustment is performed using a Proportional-Integral-Derivative (PID) controller. Control-theoretic time synchronization methods, including PID-based approaches, can improve accuracy and robustness against clock drift. For example, the research in [47] demonstrates that feedback-based synchronization achieves stable and scalable performance in WSNs, supporting the use of a PID controller for dynamic tick rate compensation.

Both high- and low-quality oscillators can be synchronized accurately with this method. The oscillator quality mainly influences the magnitude of the required compensation, not the general applicability of the method.

### 4.3. Latency in Wireless Communication Between a Host and Node

**Figure 4:**
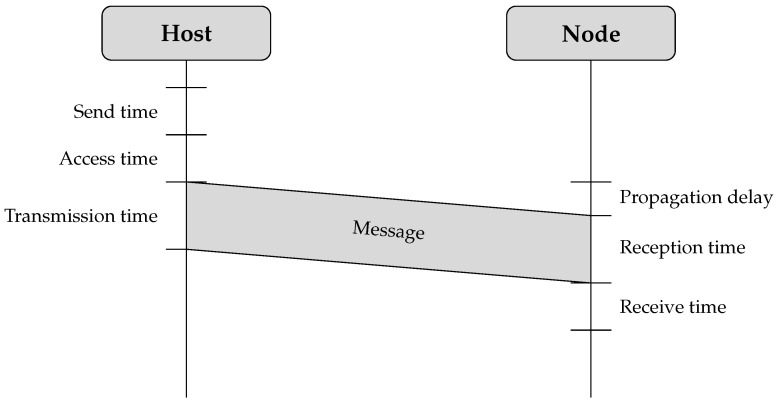
Wireless communication introduces several factors that contribute to synchronization errors.

When a host synchronizes with a GNSS module by adjusting the tick rate and replacing its RTOS ticks with the extended Unix epoch time in milliseconds, it can effectively distribute this timing information to all sensor nodes within its network. However, wireless communication introduces several sources of synchronization error, as illustrated in Figure 4. Additionally, when using an RTOS, the execution delay of the thread responsible for sending a message, including context switch latency, adds further timing uncertainty.

- Send time. This refers to the time required to construct the message and forward the send request to the MAC layer on the transmitting device. This process involves higher network layer operations, appending protocol headers, and preparing the data for transmission.
- Access time. This delay occurs while waiting for permission to use the transmission channel before the actual transmission begins. It is influenced by the specific access method implemented in the wireless system.
- Transmission time. This is the time necessary to transmit all bits of the message over the wireless medium. It depends on the total message size and the data rate.
- Propagation time. This is the time interval between when a message leaves the sender and when it reaches the receiver. This delay is largely determined by the distance between the two nodes and the speed at which electromagnetic waves propagate. However, obstacles and interference in the environment can introduce additional latency. When nodes operate within the same physical medium, propagation delays tend to be minimal and are often negligible in critical path analysis.
- Reception time. This is the period required for the receiver to fully acquire the transmitted message. It includes demodulation, signal decoding, and error correction processes.
- Receive time. This refers to the time needed to process the received message and deliver it to the application layer. This stage involves cyclic redundancy check (CRC) validation, packet reassembly, and application-layer handling.

A wide range of time synchronization methods are available to mitigate delays in wireless communication, as discussed in Section 3.1. Given that the system relies on message exchanges between a host and a node to establish network connectivity, a pairwise synchronization approach is applied, consisting of a two-message exchange between the host and each node. This approach is also employed in LTS, where it forms the basis of time synchronization in a tree-spanning topology [27]. In the method presented in this paper, the topology is limited to two levels (host and nodes), resulting in pairwise synchronization without multi-hop tree formation.

The pairwise synchronization approach estimates the clock offset from four timestamps obtained during the two-way message exchange between the host and a node. This process is discussed in more detail in Section 5.3. Under the assumption of symmetric wireless communication delays in both directions, the individual delay components are effectively compensated when calculating the time offset.

## 5. Novel RTOS-Integrated Time Synchronization Approach

The proposed time synchronization method consists of three steps, each corresponding to the determining factors discussed in Section 4.

1. Alignment of RTOS ticks with the extended epoch time in milliseconds from the GNSS module to store the Unix time in the tick count variable, thereby minimizing errors when sharing time information across the sensor network.
2. Tick rate compensation on the host using the GNSS PPS signal to accurately determine the Compare Match Value of the SysTick Timer.
3. Time synchronization of a node with the host through two complementary approaches. First, a broadcast message is used to calculate the correct Compare Match Value for the node’s SysTick Timer, ensuring accurate tick rate compensation. Second, pairwise time synchronization with nanosecond resolution timestamps is employed to eliminate the offset between the host and the individual node tick counts. Both tick counts represent the extended epoch time, and this synchronization process mitigates the errors introduced by wireless communication delays.

**Figure 5:**
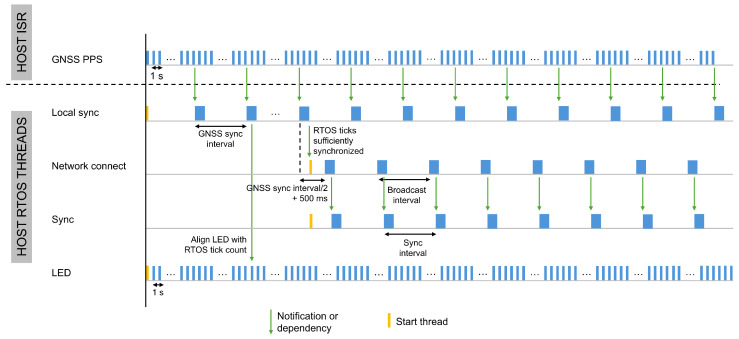
Overview of the different RTOS threads on a host used for the synchronization method and evaluation. This illustrates the start times, execution order, and dependencies of these threads over time. The dots indicate omitted time intervals during which thread executions continue.

**Figure 6:**
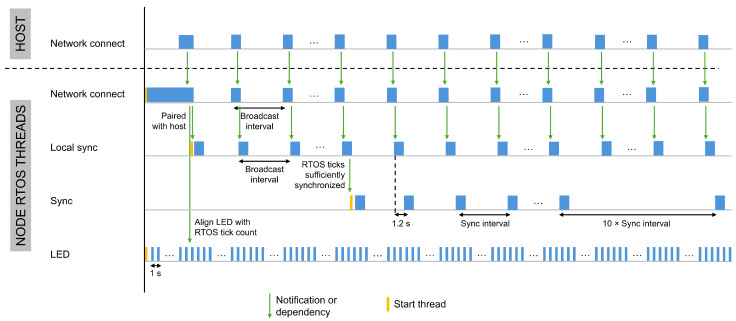
Overview of the different RTOS threads on a node used for the synchronization method and evaluation. This illustrates the start times, execution order, and dependencies of these threads over time. The dots indicate omitted time intervals during which thread executions (blue lines) continue.

Dedicated threads handle all time synchronization operations. Figure 5 and Figure 6 illustrate the start times, execution order, and interdependencies of these threads on a host and a node, respectively. Although the overall thread structure is consistent across devices, the specific roles of the threads and their execution order vary depending on the device type, as summarized in Table 1.

The Light Emitting Diode (LED) thread does not participate in the synchronization process and serves solely for evaluation purposes. This thread executes once per second to toggle a LED at what should correspond to an absolute integer second. During evaluation, we monitor the LED activation time using a logic analyzer and compute the error between the reference GNSS module and the host and sensor nodes. This measurement quantifies how accurately the host and nodes execute a task at a predefined time relative to the global reference, as discussed in Section 6.2.

The accuracy achieved with the presented synchronization method depends on the frequency at which the tick rate compensation and tick count offset are recalculated. This is determined by three factors: the number of GNSS PPS signals used by the host for tick rate compensation, the transmission rate of the broadcast message from the host to the nodes, and the frequency of synchronization messages between a node and the host. Each factor corresponds to a specific time interval. In this section, we describe the synchronization approach in a general form and introduce these intervals symbolically, without assigning numerical values. Section 6 assigns numerical values to these intervals and evaluates their impact on time synchronization accuracy. The intervals are defined as follows:

- GNSS sync interval:defines at which frequency the host reassesses the tick rate compensation mechanism. To reduce the overhead of the Local Sync thread, the system does not use every GNSS PPS signal. Instead, this interval specifies the number of GNSS PPS signals, and therefore seconds, that must elapse before performing the tick rate compensation, based on the required system accuracy. For example, with a 10 s interval, the system counts ten GNSS PPS signals before a notification is given to the Local Sync thread to calculate the tick rate compensation.
- Broadcast interval: defines how often the host sends a broadcast message to detect new sensor nodes and adjust the tick rate of known nodes. As the broadcast message also serves for tick rate compensation on the sensor nodes, this interval likewise determines how often the nodes reassess this mechanism.
- Node sync interval: defines how often a sensor node calculates, and if necessary adjusts, the error in its tick count relative to the extended epoch time on the host. A pairwise synchronization approach determines this synchronization error using a two-way exchange of nanosecond resolution timestamps between the node and the host. To minimize energy consumption, this time synchronization can be integrated with data or diagnostic transmissions, so the length of the node sync interval depends on the rate of these transmissions.

The following subsections discuss in detail the three steps of the synchronization process.

### 5.1. Align RTOS Ticks on Sensor Host with Extended Epoch Time from GNSS Module

The sensor host retrieves GPRMC messages, which are a standard GPS sentence format, from the GNSS module. These messages provide the date and UTC time, which are used to generate the epoch time intended to replace the traditional RTOS tick count. To serve as a direct replacement, the epoch time must be scaled according to the configured tick rate and resulting tick count unit.

In the presented setup, we convert the epoch time to milliseconds to match the tick count unit and replace the traditional RTOS tick count. To support this representation, we change the FreeRTOS tick count data type from uint32_t to uint64_t. This modification allows the system to maintain a millisecond-resolution epoch timestamp for hundreds of millions of years and prevents overflow of signed 32-bit Unix time representations, commonly referred to as the Year 2038 problem [48]. Increasing the tick rate, which results in a nanosecond-resolution tick count, reduces the time to overflow.

This system enables the host, and consequently all nodes, to execute tasks at precisely defined moments in time, with an accuracy of up to 1 ms. By leveraging subticks with a theoretical period of 62.5 ns when using a clock frequency of 16 MHz, it can achieve sub-millisecond precision. The actual precision attainable with subticks depends on the clock frequency and the synchronization accuracy between the GNSS module and the host.

**Figure 7:**
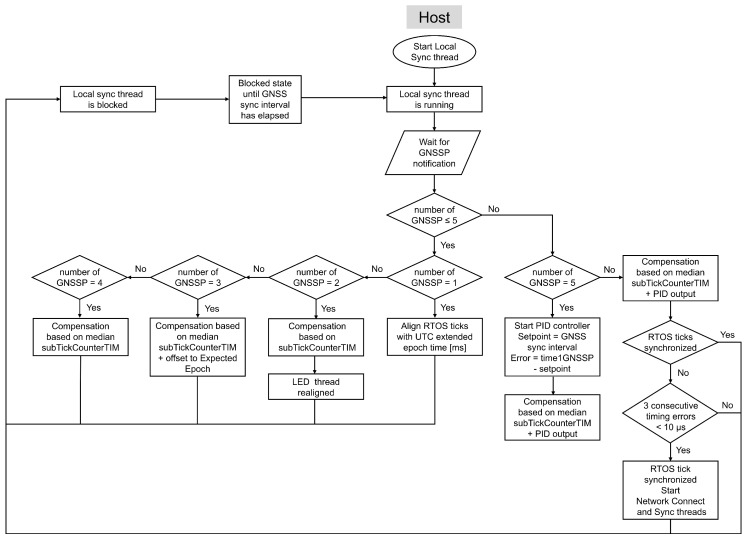
Flowchart illustrating the RTOS Local sync thread executed on the host to align RTOS ticks with the extended epoch time obtained from the connected GNSS module and calculate the tick rate compensation. The host uses an alternative computation method for the first four GNSS sync intervals. Starting with the fifth interval, the host activates a PID controller based on the difference between elapsed ticks and subticks between two GNSS sync intervals (time1GNSSP) and the GNSS sync interval. The host derives the final tick rate compensation by combining the compensation based on the mean subTickCounterTIM value with the PID controller output.

At host startup, the system initiates the Local sync thread and the LED tread, as shown in Figure 5. The Local sync thread handles the initial alignment RTOS ticks and computes the tick rate compensation on the host. Figure 7 illustrates the flowchart of this thread.

During execution, the Local sync thread waits for a notification from the GNSS PPS Interrupt Service Routine (ISR), which indicates that a GNSS sync interval has elapsed. At the first GNSS sync interval, the date and UTC time from the most recent GPRMC message are used to compute the epoch value in seconds. This value is then incremented by 2 s. The first second compensates for the fact that the most recently received GPRMC message corresponds to the previous PPS pulse and includes the processing delay required to read the message over Inter-Integrated Circuit (I^2^C) from the GNSS module. The second second ensures alignment with the moment when the epoch time will replace the tick count at the next GNSS PPS interrupt. To express the epoch value in milliseconds, it is multiplied by 1000, under the assumption that each GNSS PPS interrupt occurs precisely at the start of a new second. At the subsequent GNSS PPS, a flag is set once and is consumed during the next xTaskIncrementTick in FreeRTOS to update the tick count. Therefore, because one RTOS tick equals 1 ms, the calculated extended epoch value is incremented by 1 ms before this update.

The alignment of the RTOS ticks on the host with the extended Unix time calculated from the UTC time of the GNSS module is done only once. Afterwards the system relies on the tick rate compensation mechanism described in the following section.

### 5.2. Compensation of the Tick Rate on the Sensor Host by Using the GNSS PPS

**Figure 8:**
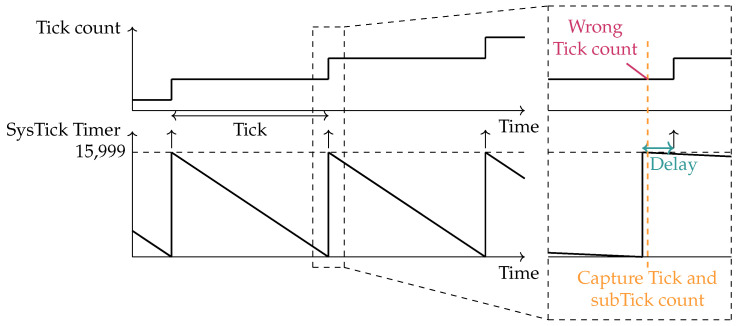
When the SysTick Timer overflows, it triggers a Tick Interrupt to increment the tick count. If the overflow occurs just before a GNSS PPS, the tick count may be captured before it is updated, resulting in an incorrect value. This is avoided by checking the overflow flag in the SysTick Control and Status Register and adding one tick to the stored value if the flag is still set.

The sensor host is connected to a GNSS module that generates a PPS signal, which acts as an interrupt source for the host. When this interrupt occurs, the system first records the remaining value of the SysTick Timer register. Because this timer counts down, the number of elapsed subticks can be calculated from the remaining value. It then stores the current tick count. Both values are required for the synchronization process. When the SysTick Timer operates at a theoretical clock frequency of 16 MHz, it updates every millisecond with a resolution of 62.5 ns. Because of this high update rate, the SysTick Timer value must be recorded first, before the tick count, which only changes every millisecond. However, if the SysTick Timer overflows and starts counting down again just before these values are read, the tick count might not have been updated yet, as illustrated in Figure 8. In this case, the system would use an incorrect tick count, leading to inaccurate synchronization. To prevent this, the system checks whether the overflow flag in the SysTick Control and Status Register is still set. This flag resets when the xTaskIncrementTick task increments the tick count. If the flag remains set, the tick count has not yet been updated, so the system adds one tick to the stored value to align it with the stored subtick count.

The tick and subtick values stored at the PPS interrupt are used for tick rate compensation on the host, ensuring minimal deviation from the extended epoch time represented in the tick count. To optimize resource usage, tick rate compensation is performed at the GNSS sync interval rather than at every GNSS PPS pulse. The impact of this interval on synchronization accuracy is discussed in Section 6.

A timer running at the same frequency as the SysTick Timer counts the number of subticks during each GNSS sync interval, subTickCounterTIM. This count is stored in a moving-window array to compute the median over the last 20 GNSS sync intervals. The median is then used to estimate the effective frequency of both timers and is incorporated into the tick rate compensation process starting from the third GNSS sync interval to compensate for the clock drift caused by clock inaccuracies. Additionally, subTickCounterTIM is used to determine the duration of a single subtick in nanoseconds by dividing the length of the GNSS sync interval by the count. Together with the values for the tick and subtick count stored at the PPS interrupt, the system calculates the RTOS time elapsed during the GNSS sync interval, time1GNSSP.

Every GNSS sync interval we calculate a new Compare Match Value for the SysTick Timer to compensate the tick rate error as initially proposed in [11]. When using a theoretical frequency of 16 MHz for the SysTick Timer, adapting the Compare Match Value allows us to perform changes in steps of theoretically 62.5 ns for an accurate tick rate. Before starting a PID controller to calculate the Compare Match Value, initial tick rate compensations are executed, as illustrated in Figure 7.

At the second GNSS sync interval an initial compensation for the deviation in tick rate is calculated. The theoretical tick rate of FreeRTOS is 1 ms, meaning the SysTick Timer counts down from 15,999 to 0 at a frequency of 16 MHz. At the end of a GNSS sync interval we expect GNSS sync interval duration [s] × 1000 × 15,999 subticks. The difference between this value and subTickCounterTIM equals the initial compensation.

Additionally, the RTOS utilizes a semaphore to signal the LED RTOS thread, prompting it to realign the LED with the start of a new RTOS second, as shown in Figure 5. This alignment facilitates monitoring of time synchronization accuracy at a later stage and will be discussed in Section 6. Upon receiving this semaphore, the LED thread calculates the required delay based on the LED interval, in this research set to one second, and the current RTOS tick count, which represents the extended epoch value in milliseconds. By taking the modulus of the tick count with the LED interval, the LED thread determines the number of milliseconds it must wait to synchronize precisely with the start of the next second, ensuring alignment with the RTOS tick count.

The tick rate compensation implemented at the third GNSS sync interval consists of three parts. First, the system calculates the difference between the expected number of subticks and the median of the last three subTickCounterTIM values. Second, it applies an offset adaptation to align the RTOS tick count, which represents the extended epoch time in milliseconds, with the expected epoch time. Third, to execute threads at a predefined time interval, it compensates for execution-time offsets, caused by for example context switching, by adjusting the LED thread to its expected execution time at the start of each UTC second. The results of these three parts are combined and separated into an integer and a fractional component. The integer part is added directly to the existing Compare Match Value of the SysTick Timer. The fractional part is accumulated across subticks until it reaches the equivalent of one full subtick, at which point it is added to the Compare Match Value at the next subtick only.

Starting from the fourth GNSS sync interval, the system computes a tick rate compensation from the difference between the expected number of subticks and the median of a moving window of the previous subTickCounterTIM values, with a maximum of 20 values. From the fifth GNSS sync interval onward, this compensation is combined with the output of a PID controller. Ideally, the duration of time1GNSSP matches that of the GNSS sync interval, which serves as the setpoint for the PID controller. The PID error is therefore defined as the difference between these two durations. The PID gain parameters were determined empirically for the custom-developed hardware.

The output of the PID controller is converted to subticks and gradually distributed over the GNSS sync interval to introduce smooth changes to the Compare Match Value. The resulting new Compare Match Value for the SysTick Timer is the combination of the previous Compare Match Value, the integer part of the PID output and the compensation calculated based on the median of a moving window of maximum 20 subTickCounterTIM values. In addition, the fractional part of the compensation is not discarded. Since the SysTickhandler is triggered every millisecond, the extra fractional adjustments are continuously accumulated. Once their sum reaches one, the Compare Match Value is modified in the next SysTickhandler execution. After this update, the Compare Match Value remains set to the integer part of the PID output compensation, until the sum of fractional adjustments reaches one again, at which point the process repeats. To reduce the update rate of the Compare Match Value, the fractional part is evaluated before a new Compare Match Value is applied. If the fractional part is higher than 0.5, one subtick will be added to the compensation and subtracted from the fractional part. This adjustment ensures that the remaining fractional part is always smaller than 0.5. As a result, the sum of fractional adjustments requires multiple milliseconds to reach one subtick, which reduces the frequency of Compare Match Value updates while preserving the overall compensation.

If the difference between the duration of time1GNSSP and that of the GNSS sync interval remains below a threshold defined according to the accuracy requirements of the target application for three consecutive measurements, the RTOS ticks are considered sufficiently synchronized with the GNSS PPS signal. This threshold was set to 10 μs in the experimental evaluation. At this stage, the Network Connect and Sync thread are started on the host, as illustrated in Figure 5 and Figure 7. The Network Connect thread is responsible for discovering sensor nodes, while the Sync thread listens for synchronization requests from the nodes.

### 5.3. Time Synchronization of a Sensor Node with a Sensor Host

The Network Connect thread is responsible for discovering new sensor nodes and integrating them into the network managed by the initiating host. This thread is only activated once the host has achieved sufficient synchronization with the GNSS module, as detailed in Section 5.1. Upon initialization, the network thread is aligned with the GNSS sync interval to prevent interference between processes and is deliberately scheduled to execute precisely in between two GNSS sync intervals with an additional offset of 500 ms to avoid conflicts with the GNSS PPS and the execution of the LED thread every second, as illustrated in Figure 5. The initial execution time of the network thread is calculated based on the current tick count. After that, the host’s enhanced tick rate enables deterministic and more accurate execution of the network thread, which in turn allows broadcast messages for node discovery to be transmitted more precisely at the broadcast interval.

**Figure 9:**
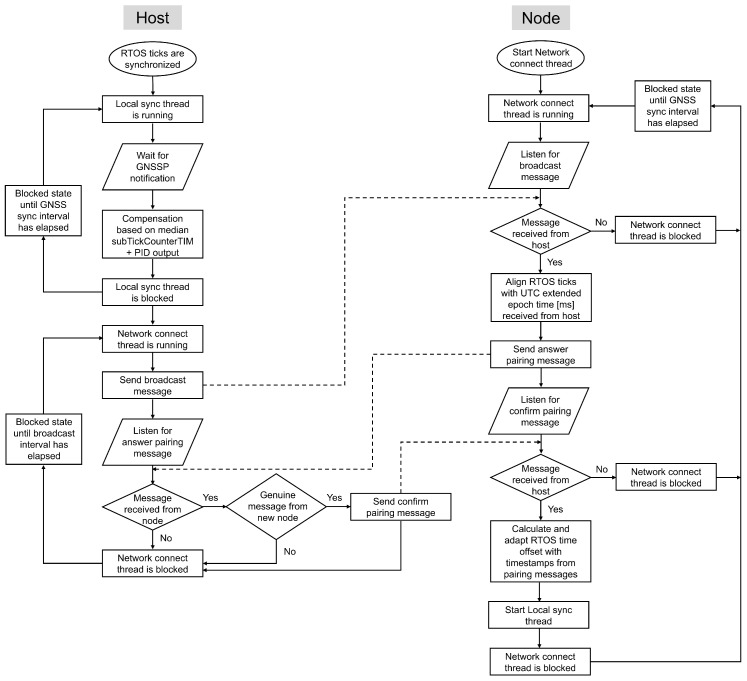
Flowchart illustrating the RTOS Network connect thread running on both the host and the node for node discovery, pairing, and initial time synchronization. The host periodically transmits broadcast messages that enable discovery of new nodes and support tick rate compensation for paired nodes. Upon receiving a valid broadcast message, a new node aligns its RTOS ticks with the extended epoch time and initiates the pairing exchange. During the pairing message exchange, the host and node record timestamps to estimate the initial time offset.

Besides facilitating the connection of sensor nodes to the sensor host, the Network Connect thread also plays a crucial role in synchronizing a sensor node with its host. Since a sensor node is not directly connected to a GNSS module, it depends on the host to compensate for its tick rate and to replace its tick count with the extended epoch time. Figure 9 shows the flowchart of the Network connect thread running on both the host and the node during node discovery, pairing, and initial time synchronization.

#### 5.3.1. Node Discovery and Saving Epoch Time in the RTOS Tick Count

When a sensor node is powered on, the system starts the Network connect thread and the LED thread, as shown in Figure 6. The Network connect thread manages the one-time pairing process and initial alignment of RTOS ticks with the host. After pairing, the thread remains active to receive broadcast messages from the host, which the node uses to compute the tick rate compensation. Upon receiving the first broadcast, the node extracts the extended epoch time in nanoseconds from the message and uses it to replace its own RTOS tick count. Because this extended epoch time corresponds to the RTOS tick count on the host just before the message was sent and the wireless transmission process introduces several delays, a small offset will initially exist between the tick count of the host and the node.

#### 5.3.2. Initial Time Offset Compensation on a Node

To eliminate this offset, a pairwise synchronization approach is applied, using timestamps recorded during the commissioning process on both the sensor host and the sensor node. The clock offset is estimated using a two-way message exchange (four timestamps), as employed in LTS, and requires only two messages [27], making it well-suited for low-power sensor nodes. This approach enables compensation of symmetric wireless communication delays, thereby ensuring precise time alignment between the host and the sensor node, as discussed in Section 4.3.

**Figure 10:**
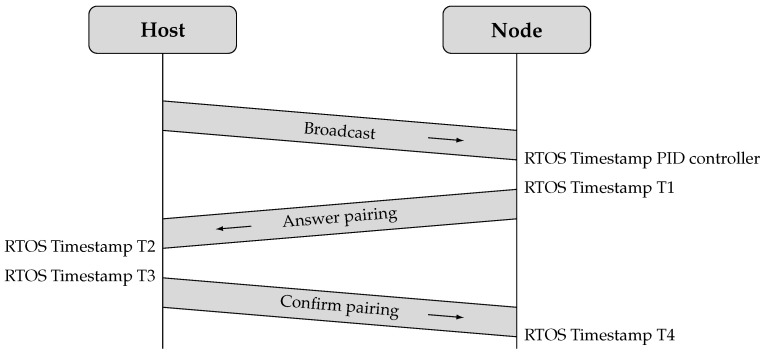
Three-way communication between host and node, used for node discovery, initial pairing, and timestamp exchange for synchronization. After the initial pairing, a node uses the broadcast message as a reference to calculate the tick rate compensation.

The synchronization step is integrated into the discovery and pairing process of new sensor nodes to minimize overhead. As illustrated in Figure 10, this process consists of a three-way message exchange between the host and the node. Upon receiving a broadcast message from the host, a node responds to initiate pairing. When preparing the answer pairing message, the node records a first timestamp in nanoseconds as close as possible before transmission. This timestamp combines the RTOS tick count in milliseconds with the current SysTick Timer value. The node embeds this timestamp in the message together with a placeholder for a second timestamp, producing a data packet that matches the size of the host’s response. The host’s response includes two timestamps, which the node uses to calculate the offset. This method improves the accuracy of timestamp recording and increases the precision of the offset calculation.

When the host receives the answer pairing message from the node, it records a second timestamp in nanoseconds. The host then confirms the pairing and completes the LTS synchronization process by sending a confirmation message to the node. This message includes two nanosecond resolution timestamps: the second timestamp recorded upon receiving the pairing message and a third timestamp recorded as close as possible before transmitting the confirmation.

Upon receiving the host’s confirmation message, the node records a fourth timestamp in nanoseconds. With this step, all four timestamps are collected under the same conditions, enabling a more accurate calculation of the time offset. The node applies this offset to adjust its tick count, thereby synchronizing with the host’s extended epoch time. After completing this synchronization step, the node starts the Local sync thread.

#### 5.3.3. Tick Rate Compensation on a Node

**Figure 11:**
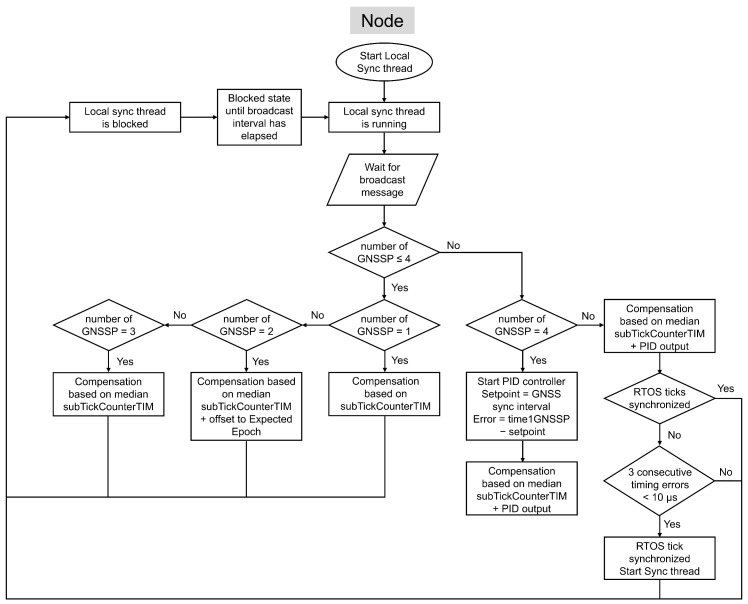
Flowchart illustrating the RTOS Local sync thread running on the node after pairing with the host, where the node calculates tick rate compensation using broadcast messages. The node uses an alternative computation method for the first three broadcast messages. Starting with the fourth broadcast message, the node activates a PID controller based on the difference between elapsed ticks and subticks between two broadcasts (time1BC) and the broadcast interval. The node derives the final tick rate compensation by combining the compensation based on the mean subTickCounterTIM value with the PID controller output.

After the node initially synchronizes its tick count with the host, it relies on broadcast messages to compensate for tick rate errors, which are handled by the Local sync thread. The node receives these messages at the broadcast interval, which replaces the GNSS sync interval used by the host. As on the host, the node applies a sequence of initial tick rate compensations before activating the PID controller, as illustrated in Figure 11.

At the first Broadcast interval after pairing, the node computes an initial tick rate compensation based on the subTickCounterTIM. At the second Broadcast interval, the node applies a compensation based on the same three components used on the host: the mean of the subTickCounterTIM, the offset to the expected epoch time, and the offset associated with the LED thread. Starting from the third Broadcast interval, the node derives the tick rate compensation from the difference between the expected number of subticks and the median of a moving window of maximum 20 subTickCounterTIM values. From the fourth Broadcast interval onward, the system combines this compensation with the output of a PID controller that compares the accumulated RTOS ticks and subticks (time1BC) with the expected duration of the broadcast interval. When a node receives a broadcast message, it takes a timestamp of the current RTOS ticks, as shown in Figure 10. The resulting compensation is corrected by adjusting the SysTick Timer’s Compare Match Value in the same way as the host.

However, an additional timing error must be considered on the node side. Ideally, the broadcast should be received exactly at the broadcast interval. Any deviation in the host’s transmission timing introduces an error into the node’s compensation process. To correct for this, the timestamp embedded in the broadcast message is used to estimate the host-side transmission error, and this error is incorporated into the PID-based tick rate compensation mechanism on the node.

#### 5.3.4. Start Sync Thread for Time Offset Compensation

**Figure 12:**
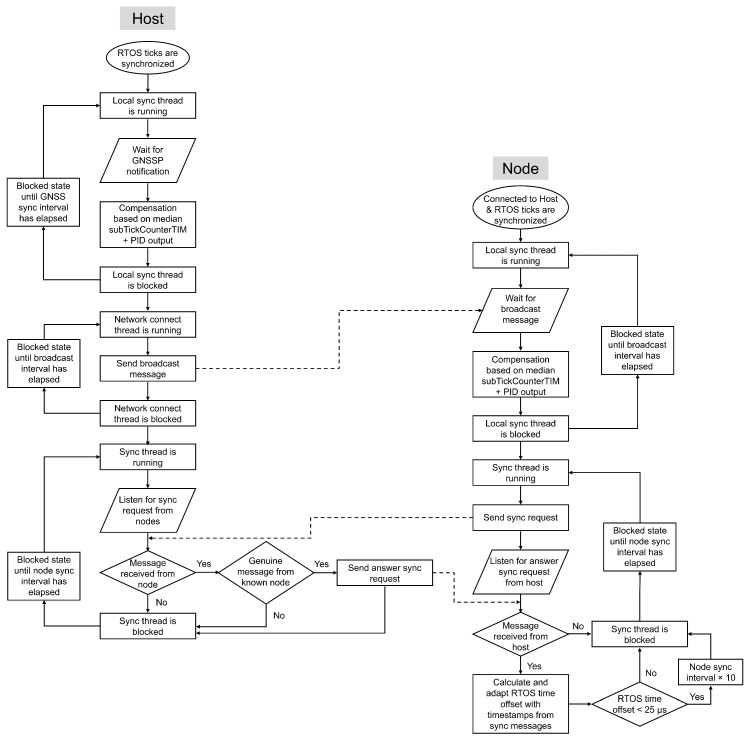
Flowchart illustrating the RTOS Sync thread executed on both the host and the node after pairing, when RTOS ticks are sufficiently synchronized and the host has not received a pairing response from a new node. In this phase, the node sends a sync request to the host to align its RTOS ticks with the extended epoch time using a two-way message exchange with timestamping to calculate the offset. The Sync thread interval is increased to 10 times its initial value once the offset falls below a threshold defined according to the accuracy requirements of the target application. This threshold was set to 10 μs in the experimental evaluation.

If the difference between the duration of time1BC and that of the GNSS sync interval remains below a synchronization threshold (set to 10 μs in the experimental evaluation) for three consecutive measurements, the RTOS ticks are considered sufficiently synchronized. At this stage, the node starts the Sync thread, as illustrated in Figure 6 and Figure 11. Figure 12 shows the flowchart of this thread on both the host and the node, along with its scheduling relative to the Local sync and Network connect threads.

The Sync thread repeats the LTS synchronization process to compute the offset between the node’s and the host’s RTOS ticks. The offset, which reflects the difference in extended epoch time between host and node, is critical for executing tasks at precise moments in time. The synchronization thread executes 1.2 s after every broadcast interval: the node sends a sync request to the host, the host replies, and four nanosecond-resolution timestamps are obtained to calculate the offset. The execution time of the Sync thread is increased to 10 times the broadcast interval, when the absolute mean of the moving window of the calculated offset of the LTS based two-way synchronization is below a threshold defined according to the accuracy requirements of the target application. The threshold was set to 25 μs in the experimental evaluation.

In the presence of communication interference leading to the loss of broadcast synchronization messages, nodes continue to apply the previously determined tick rate compensation. This allows the local clock to maintain a reasonable time estimate for a limited duration. If a large number of consecutive broadcasts is lost, the accumulated time error may prevent correct reception of subsequent synchronization messages. In such cases, the node actively executes the Sync thread and periodically sends synchronization requests to the host until a response is received. The time offset is then corrected, after which normal operation resumes.

## 6. Evaluation of the Proposed Time Synchronization Method

We implemented the proposed method outlined in Section 5 using the hardware described in Appendix A. Note that the proposed method is independent of the hardware used in this evaluation. The setup consisted of a GNSS module connected to the host and four nodes to demonstrate the accuracy of the time synchronization method, as illustrated in Figure 2. We evaluated the proposed method with respect to two aspects: (1) tick rate compensation and (2) task execution time across the WSN. For both aspects, we varied the broadcast interval and clock frequency across multiple configurations. Table 2 summarizes the experimental configurations and indicates the figures in which the corresponding results are presented.

### 6.1. Evaluation of the Tick Rate Compensation

Both the host and the nodes implement compensation mechanisms to correct tick rate deviations caused by crystal oscillator inaccuracies, thereby enabling more precise execution of RTOS tasks. The host computes its compensation based on the GNSS sync interval, whereas the nodes derive their compensation from the broadcast interval, as discussed in Section 5. Each device quantifies its tick rate deviation by comparing the measured number of ticks (in milliseconds) and subticks (in nanoseconds) during a synchronization interval with the nominal interval duration. As a baseline reference, prior to applying tick rate compensation, the host exhibited a timing error of approximately 260 μs over a 10 s GNSS sync interval in our experimental setup. The results obtained after enabling tick rate compensation are presented next and demonstrate the resulting improvement in timing accuracy.

We conducted experiments using two different durations for the GNSS sync and broadcast intervals: 10 s and 60 s. This configuration allowed us to evaluate the effect of more frequent tick rate compensation on the timing errors of these intervals. Longer intervals reduce the number of broadcast receptions required by each node, thereby lowering overall energy consumption. In addition, we assessed the impact of a higher clock frequency on both the host and nodes. Increasing the clock frequency supports faster task execution and is expected to reduce the timing error.

**Figure 13:**
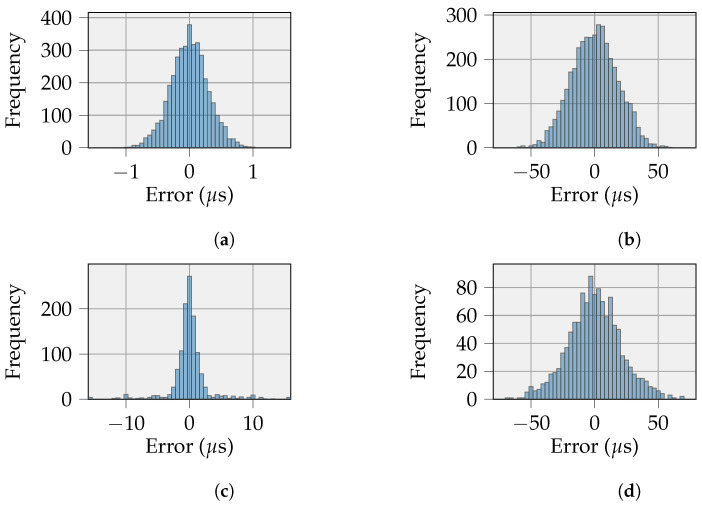

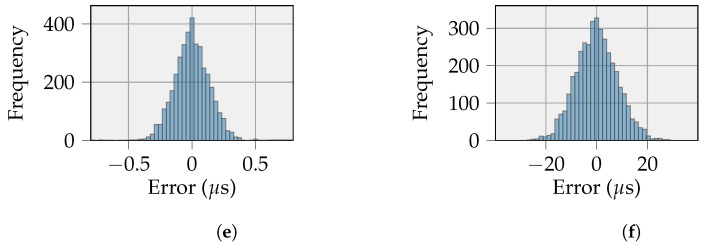
Timing error on the duration of a GNSS sync interval on a host and a broadcast interval for one of the 4 nodes in the experimental setup. (**a**) Host: GNSS sync interval = 10 s, clock frequency = 16 MHz; (**b**) Node: broadcast interval = 10 s, clock frequency = 16 MHz; (**c**) Host: GNSS sync interval = 60 s, clock frequency = 16 MHz; (**d**) Node: broadcast interval = 60 s, clock frequency = 16 MHz; (**e**) Host: GNSS sync interval = 10 s, clock frequency = 32 MHz; (**f**) Node: broadcast interval = 10 s, clock frequency = 32 MHz.

Figure 13 presents the distribution of the PID controller output for the host and a representative node after tick rate compensation under each tested interval and clock frequency configuration. We report results for a single node because all four nodes exhibited comparable timing behavior. The plots show the timing error for different durations of the GNSS sync interval on the host and for different values of the broadcast interval on the nodes, measured over 10 h of continuous operation. Smaller timing errors indicate a more accurate RTOS tick duration and consequently improved timing precision across the system. The following comments and conclusions can be drawn from the different experiments:

- The proposed tick rate compensation method substantially reduces the timing error on the interval duration for both the host and the nodes, compared with operation without compensation. The higher timing error observed on the nodes can be attributed to the broadcast message used for tick rate compensation, which is affected by both the host’s context switch time and the non-deterministic delay of the one-way wireless communication.
- Increasing the interval duration slightly broadens the synchronization error distribution for both the host and the node. At the host, the longer interval increases the probability of larger timing errors; however, the distribution remains strongly concentrated around zero, indicating that most timing errors remain small. At the node, increasing the interval duration alters the distribution shape, resulting in a flatter central region and a wider spread of timing errors, although the highest concentration of samples remains centered around zero. Despite the increased dispersion of timing errors, the persistence of a dominant peak at zero confirms that effective synchronization is maintained even at extended intervals, thereby supporting the suitability of the proposed approach for low-power IoT devices.
- Increasing the clock frequency further reduces the timing error on both the host and the nodes, with the magnitude of improvement corresponding to the frequency scaling factor. A higher clock frequency enables smaller context switch time and faster task execution.

### 6.2. Evaluation of Task Execution Time Across the WSN

The goal of the proposed synchronization method is to use the extended epoch time as a common time source across the host and nodes, enabling simultaneous and more accurate execution of tasks at predefined moments. A LED thread is executed on the host and nodes in the experimental setup with the same priority to quantify the error between the execution times. This thread should be executed every second and turns the LED on and off. We evaluate the execution time error using a Logic Analyzer that records when a LED turns on at the GNSS module, the host, and four nodes. Each LED is expected to turn on once per second and simultaneously across all devices, with the GNSS module serving as the time reference. We select the GNSS module as the reference because its LED toggles at an absolute integer second, providing a precise temporal anchor.

**Figure 14:**
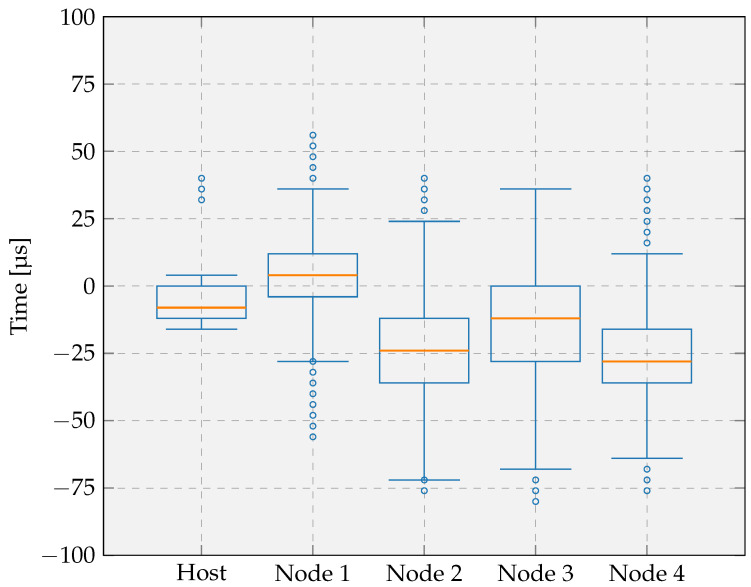
Synchronization error of the host and sensor nodes relative to the GNSS reference during 10 h of continuous operation with a clock frequency of 16 MHz and a broadcast interval of 10 s. The median is shown in orange, and outliers are indicated by blue circles.

The achievable synchronization accuracy depends on the GNSS sync interval, the broadcast interval, the node sync interval, and the clock frequency of the host and nodes. Figure 14 shows the synchronization error of the host and nodes, operating at a 16 MHz clock frequency, relative to the GNSS module. All intervals were initially set to 10 s, and the system was evaluated over 10 h of continuous operation. The node sync interval is increased to 10 times the broadcast interval, which is 100 s in our setup, when the absolute mean of the moving window of the calculated offset of the LTS based two-way time synchronization is less than 25 μs. Each boxplot represents the median, interquartile range, and outliers calculated of the difference between the time when the LED switches on at the host or nodes and when it switches on at the GNSS module.

The results indicate that the host exhibits the lowest variability, which is expected based on the results discussed in Section 6.1 and given the wired connection to the GNSS module. The timing error distribution is centered close to zero, with a median of −8 μs, showing that 50% of the measurements remain within 12 μs of the ideal value. The whiskers extend from −20 μs to 4 μs, demonstrating low variability. Only a few outliers fall outside this range, suggesting that the synchronization method occasionally introduces larger deviations. Overall, the results indicate high timing precision with a small systematic bias toward negative error, indicating a slight remaining offset. This offset arises because, on the host, the LED activation is performed within an RTOS thread, and the associated context switch delay introduces a minor timing deviation relative to the GNSS module’s LED.

The nodes show comparable performance, although with slightly higher median errors and a larger spread, consistent with the findings in Section 6.1 and the additional variability introduced by the wireless communication. Their medians range from −28 μs to 4 μs, and the interquartile ranges extend approximately from −36 μs to 12 μs. The whiskers lie between −72 μs and 36 μs, showing that the timing error remains limited and consistent across all nodes. These stable results over 10 h of continuous operation demonstrate that, even under increased variability, the synchronization method maintains high accuracy and robust network-wide behavior. Importantly, the timing error remains well below 100 μs while relying solely on wireless broadcast messages from the host, eliminating the need for a GNSS and RTC module on each node.

**Figure 15:**
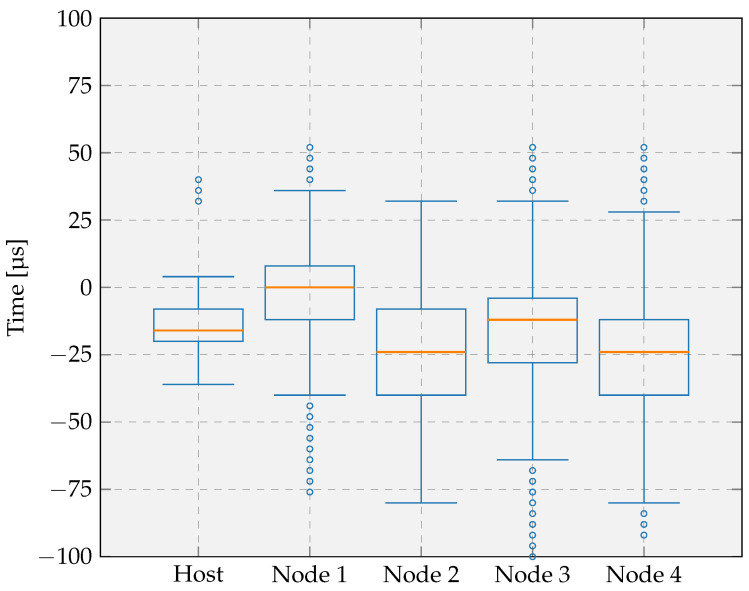
Synchronization error of the host and sensor nodes relative to the GNSS reference during 10 h of continuous operation with a clock frequency of 16 MHz and a broadcast interval of 60 s. The median is shown in orange, and outliers are indicated by blue circles.

We repeated the experiment using longer GNSS sync, broadcast and node sync intervals to evaluate the impact of reducing the time synchronization frequency while maintaining a 16 MHz clock frequency. The initial intervals are increased to 60 s, and the node sync interval was further extended to 600 s when the time offset between the host and node remained below 25 μs. The resulting timing performance, shown in Figure 15, shows similar characteristics to those obtained with the shorter interval configuration. The medians fall between −24 μs and 0 μs, demonstrating a consistent and small bias comparable to the previous results. The interquartile ranges, spanning from approximately −40 μs to 8 μs, remain of similar magnitude to those observed with the shorter interval, indicating that the increased interval duration has only a minor impact on variability. The whiskers extend from −80 μs to 36 μs, which reflects only a limited increase in the overall spread. The last two devices exhibit slightly more outliers than with the shorter interval. However, these outliers remain infrequent and do not affect the general performance assessment. Altogether, these results confirm that doubling the synchronization interval introduces only a small increase in timing error variation. The synchronization method therefore remains robust and accurate even with reduced update frequency, reinforcing its suitability for low-power IoT deployments where longer intervals help minimize energy consumption.

**Figure 16:**
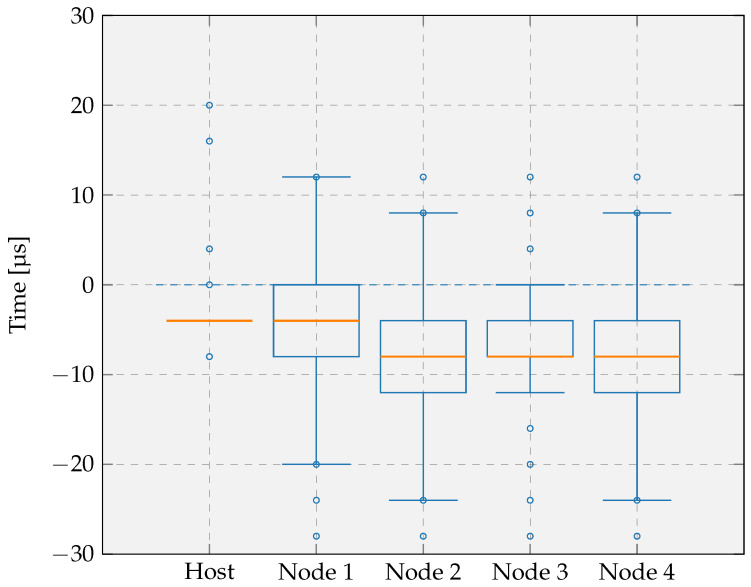
Synchronization error of the host and sensor nodes relative to the GNSS reference during 10 h of continuous operation with a clock frequency of 32 MHz and a broadcast interval of 10 s. The median is shown in orange, and outliers are indicated by blue circles.

Finally, we investigated the influence of faster processing by repeating the experiment with the synchronization intervals again set to 10 s, but with the host and nodes operating at an increased clock frequency of 32 MHz. The resulting boxplots, shown in Figure 16, demonstrate a clear improvement in timing precision compared with the initial configuration operating at 16 MHz. The medians now cluster tightly between −8 μs and −4 μs, indicating that the synchronization error is closer to zero and more uniform across devices. The interquartile ranges have narrowed significantly, with the majority of samples remaining within 8 μs of the median, whereas the original configuration exhibited spreads of up to 48 μs. For the host and Node 3, the interquartile range is not clearly visible, as the first quartile, median, and third quartile coincide or are very close due to the low variability of the synchronization error, indicating highly stable synchronization performance. The whiskers extend only from −24 μs to 12 μs, demonstrating that timing deviations are both reduced and tightly bounded. These results show that increasing the clock frequency substantially enhances synchronization accuracy and consistency across all nodes, confirming faster task execution directly benefits time alignment throughout the network.

The following comments and conclusions can be drawn from the different experiments:

- With a broadcast interval of 10 s and a 16 MHz clock frequency, the task execution error on both the host and the nodes relative to the GNSS reference remains well below 100 μs.
- The remaining offset can be attributed to the fact that both the host and the nodes toggle their LED through an RTOS thread, whereas the GNSS module does not. The involvement of a scheduled thread introduces a context switch delay, resulting in a slight timing shift. This observation also aligns with the findings from the tick rate compensation evaluation, where similar effects of thread scheduling were identified.
- Increasing the broadcast interval has only a minor effect on the execution time error, which suggests that the interval could be extended further to reduce energy consumption.
- A higher clock frequency has a significant positive effect on task-execution accuracy, reducing the timing error to within approximately ±30 μs. This improvement is expected, as faster task execution lowers the impact of scheduling delays and is consistent with the results from the tick rate compensation evaluation.

This evaluation quantifies how accurately the host and sensor nodes execute a task at a predefined time relative to a global reference. If the focus were instead placed on the relative timing error between the host and nodes during coordinated measurements across the network, the resulting errors would be smaller, as already suggested by the results shown in Figure 14, Figure 15 and Figure 16. However, such a relative evaluation is beyond the scope of this paper.

### 6.3. Comparison with Existing RTOS-Based Time Synchronization Methods

To the best of our knowledge, the only work addressing time synchronization within RTOSs is the GNSS-based system time synchronization method presented in [11,12]. This approach compensates for tick rate, tick phase, and system time using the PPS signal of a GNSS module connected to each device. The compensation is applied by adjusting the tick interval, i.e., the Compare Match value of the hardware timer. However, the use of a control strategy such as a PID controller is not explicitly described.

Synchronization accuracy is evaluated by measuring the time difference between output signal toggles generated by hardware timer compare events on different devices. Reported maximum time differences are 18 μs in [11] and 10 μs in [12], both obtained on microprocessors operating at 20 MHz.

In contrast, the method proposed in this paper extends this approach in several aspects. First, it incorporates a PID controller for tick rate compensation. Second, it eliminates the need for a GNSS module on each device by distributing time from a single GNSS-enabled host using the LTS synchronization method, thereby mitigating errors introduced by wireless communication. Third, instead of merely correcting system time, the method maps the RTOS tick count directly to extended Unix time in milliseconds, providing each device with an accurate notion of UTC time without requiring a RTC.

Two aspects are evaluated: tick rate compensation and task execution timing. The latter is most comparable to the system time evaluation in [11,12]. Task execution accuracy is assessed by scheduling a periodic task at absolute UTC second boundaries across multiple devices. The results show a maximum time difference below ±100 μs at 16 MHz and approximately ±30 μs at 32 MHz.

Overall, although the achieved accuracy is slightly lower than that of per-device GNSS-based solutions, it remains sufficiently precise for practical embedded applications. The main advantage is the reduction in hardware cost and system complexity, achieved by eliminating the need for both GNSS modules and RTCs on individual nodes.

## 7. Conclusions and Future Work

In this work, we presented a novel RTOS-integrated time synchronization approach for WSNs that enables the accurate network-wide distribution of an absolute UTC time reference using only a single GNSS-enabled host. The proposed method combines established synchronization mechanisms, including the tick synchronization approach proposed in [12], a PID-based correction mechanism for clock adjustment, and a pairwise synchronization approach based on two-way message exchange to estimate the time error introduced by wireless communication.

A key contribution of this work is the integration of the extended Unix epoch time directly into the RTOS time base, allowing the RTOS tick count to represent globally synchronized UTC time rather than only relative system uptime. This design enables the operating system scheduler and application tasks to operate directly on absolute timestamps and execute time-critical tasks at precisely defined global times. Consequently, sensor nodes can maintain a notion of UTC time and participate in synchronized network operation without requiring dedicated RTC or GNSS hardware on each node, thereby reducing hardware complexity and system cost.

By integrating time synchronization into an RTOS, tasks can be executed with precise, coordinated timing across nodes, enhancing determinism, predictability, and system-wide consistency. This capability enables synchronized data collection, real-time control, optimized channel utilization, and efficient coordination among distributed devices, which is particularly valuable in IoT applications. By combining deterministic execution with scheduled task initiation, RTOS-based systems achieve both real-time responsiveness and absolute timing accuracy, enhancing efficiency, safety, and automation in domains where precise synchronization is critical.

The proposed approach was implemented on custom-developed hardware and experimentally evaluated, demonstrating accurate tick-rate synchronization and consistent execution of time-critical tasks across the WSN. These results highlight the suitability of the method for distributed sensing applications that require precise time coordination. Such applications include synchronized sensing, including distributed acoustic localization, and seismic monitoring, which rely on accurate time alignment across the network.

As future work, the synchronization frequency at the nodes can be adaptively reduced to lower energy consumption while maintaining timing accuracy. While the host periodically performs GNSS synchronization and broadcasts updates, nodes can initially synchronize frequently to achieve accurate alignment and subsequently increase their listening interval once the timing error falls below a defined threshold. This adaptive approach enables fast initial synchronization while reducing wireless activity and energy consumption over time.

In addition, the current node synchronization mechanism can be integrated into regular data communication. By embedding timestamps in data packets and acknowledgments, clock offset estimation can be performed without a dedicated synchronization thread. This can reduce communication overhead and further improves energy efficiency.

In this work, we proposed a novel time synchronization method, which forms a key building block for a self-deployable WSN. The synchronization mechanism serves as the temporal foundation that enables autonomous coordination and communication among sensor nodes.

In future work, we aim to extend the exemplary setup used to evaluate the synchronization method toward a complete self-deployable WSN framework. The current prototype already implements several fundamental components required for such a system, including device discovery, pairing, and network-wide time synchronization. Further extensions will enable further evaluation of how the proposed synchronization protocol supports autonomous sensor network deployment and will help identify opportunities for future improvements.

## Figures and Tables

**Table 1:** Overview of the threads operating at a host and a node.

Thread	Host	Node
Local Sync	Alignment of RTOS ticks with the extended epoch time and calculation of tick rate compensation for the Systick Timer.	Calculation of tick rate compensation for the Systick Timer.
Network Connect	Discovery and pairing of new nodes using broadcast messages.	Pairing with a host and alignment of RTOS ticks with the extended epoch time, followed by reception of broadcast messages for tick rate compensation.
Sync	Reception of sync requests from nodes and transmission of timestamped responses for time offset estimation.	Initiation of sync requests to the host and computation of the time offset using four timestamps.
LED	LED toggling every second to evaluate synchronized task execution across different nodes.	LED toggling every second to evaluate synchronized task execution across different nodes.

**Table 2:** Experimental configurations and corresponding results used to evaluate the proposed time synchronization method. The GNSS sync and broadcast interval define the execution frequency of the synchronization procedure. For the task execution time evaluation, the node sync interval initially equals the broadcast interval and is increased to 10 times the broadcast interval during operation.

Evaluation	GNSS Sync & Broadcast Interval [s]	Node Sync Interval [s]	Clock Frequency [MHz]	Result
Tick rate compensation	10	/	16	Host: Figure 13a Node: Figure 13b
	60	/	16	Host: Figure 13c Node: Figure 13d
	10	/	32	Host: Figure 13e Node: Figure 13f
Task execution time	10	10–100	16	Figure 14
	60	60–600	16	Figure 15
	10	10–100	32	Figure 16

## Data Availability

The data presented in this study are included in the article and its Supplementary Materials. Further inquiries can be directed to the corresponding author.

## Supplementary Materials

The following supporting information can be downloaded at: https://www.mdpi.com/article/10.3390/s26072121/s1. Supplementary Software S1. Software implementing the proposed time synchronization method and used for the presented evaluation. The software was developed using STM32CubeIDE (STMicroelectronics, Plan-les-Ouates, Switzerland) and targets the hardware described in Appendix A.

## Abbreviations

The following abbreviations are used in this manuscript:

CRC	cyclic redundancy check
CSMA/CA	Carrier Sense Multiple Access with Collision Avoidance
FTSP	Flooding Time Synchronization Protocol
GNSS	Global Navigation Satellite System
GPS	Global Position System
I^2^C	Inter-Integrated Circuit
IoT	Internet of Things
ISM	industrial, scientific and medical
ISR	Interrupt Service Routine
LED	Light Emitting Diode
LoRa	Long Range
LTS	Lightweight Tree-based Synchronization
MAC	Medium Access Control
MCU	microcontroller unit
NTP	Network Time Protocol
OS	Operating System
PID	Proportional-Integral-Derivative
PPS	Pulse Per Second
RBS	Reference Broadcast Synchronization
RTC	Real-Time Clock
RTOS	Real-Time Operating System
TDD	Time Division Duplex
TDMA	Time Division Multiple Access
TPSN	Timing-sync Protocol for Sensor Networks
UTC	Coordinated Universal Time
UWB	Ultra-Wideband
WSN	Wireless Sensor Network

## Appendix A

This appendix provides a list of main components, including manufacturer type numbers and key specifications, to enable reproduction of the experimental setup presented in this work.

**Table A1:** List of main components and type numbers used in the experimental setup.

Category	Manufacturer	Type Number	Key Specifications
Microcontroller	STMicroelectronics, Plan-les-Ouates, Switzerland	STM32WBA52CG	Arm Cortex-M33
Crystal	NDK, Tokyo, Japan	CHPCIS3-32M	32 MHz crystal for microcontroller
Wireless transceiver	Semtech Corporation, Camarillo, CA, USA	SX1280	LoRa 2.4 GHz transceiver
Crystal	NDK	CS07103-52M	52 MHz crystal for wireless transceiver
Antenna	TE Connectivity, Schaffhausen, Switzerland	ANT-2.4-MSA-TH1	2.4 GHz antenna
Voltage regulator	Texas Instruments Incorporated, Dallas, TX, USA	TPS62740DSSR	Voltage regulator for 1.8 V
GNSS module	STMicroelectronics	X-NUCLEO-GNSS1A1	GNSS expansion board based on Teseo-LIV3F module for STM32 Nucleo
